# AAA-ATPases in Protein Degradation

**DOI:** 10.3389/fmolb.2017.00042

**Published:** 2017-06-20

**Authors:** Ravikiran S. Yedidi, Petra Wendler, Cordula Enenkel

**Affiliations:** ^1^Department of Biochemistry, University of TorontoToronto, ON, Canada; ^2^Department of Biochemistry, Institute of Biochemistry and Biology, University of PotsdamPotsdam, Germany

**Keywords:** AAA, ATPase, proteasome, protein folding, proteolysis

## Abstract

Proteolytic machineries containing multisubunit protease complexes and AAA-ATPases play a key role in protein quality control and the regulation of protein homeostasis. In these protein degradation machineries, the proteolytically active sites are formed by either threonines or serines which are buried inside interior cavities of cylinder-shaped complexes. In eukaryotic cells, the proteasome is the most prominent protease complex harboring AAA-ATPases. To degrade protein substrates, the gates of the axial entry ports of the protease need to be open. Gate opening is accomplished by AAA-ATPases, which form a hexameric ring flanking the entry ports of the protease. Protein substrates with unstructured domains can loop into the entry ports without the assistance of AAA-ATPases. However, folded proteins require the action of AAA-ATPases to unveil an unstructured terminus or domain. Cycles of ATP binding/hydrolysis fuel the unfolding of protein substrates which are gripped by loops lining up the central pore of the AAA-ATPase ring. The AAA-ATPases pull on the unfolded polypeptide chain for translocation into the proteolytic cavity of the protease. Conformational changes within the AAA-ATPase ring and the adjacent protease chamber create a peristaltic movement for substrate degradation. The review focuses on new technologies toward the understanding of the function and structure of AAA-ATPases to achieve substrate recognition, unfolding and translocation into proteasomes in yeast and mammalian cells and into proteasome-equivalent proteases in bacteria and archaea.

## Overview

Proteins are synthesized during translation through ribosomes and eliminated by degradation through proteases. Since protein synthesis and degradation are expensive ATP-consuming processes, highly selective mechanisms ascertain that only proteins allotted to degradation are eliminated. If the regulation of protein homeostasis fails, futile cycles of protein synthesis and turnover will ruin the economic budget of our cells. Functional proteins would be depleted and non-functional proteins would accumulate in cytotoxic aggregates (Kopito, 2000; Ciechanover and Brundin, 2003; Goldberg, 2003; Schmidt and Finley, 2014).

Thus, functional proteins must be sorted from non-functional proteins to meet the actual cellular situation with rapid adjustments to metabolic changes or environmental stress. How protein textures shift in response to cellular changes is an interesting question in the field of regulated protein homeostasis but out of the scope of this review. Here, we will focus on ATPases associated with diverse cellular Activities (AAA) that collaborate with proteasomes, the most complex proteases with unique opportunities for regulation of cellular proteolysis. AAA-ATPases typically convert the energy of ATP hydrolysis into mechanical force through conformational changes in their subunits, cope with the unfolding of protein substrates and synergistically act with proteasomes and proteasome-like proteases for degradation (Schmidt et al., 1999; Sauer and Baker, 2011; Matyskiela and Martin, 2013). However, they can also aid protein refolding allowing partial proteolysis or the escape of specific proteins from degradation.

A myriad of proteins is subject to AAA-ATPase coupled protein degradation by proteasomes. Proteasomal substrates are short-lived, have crucial functions within a short time frame and are eliminated within few minutes by proteasomal proteolysis. Proteasomal substrates are usually post-translationally modified by poly-ubiquitin chains, a series of ubiquitin molecules linked by isopeptide bonds to each other and to the substrate. The first proteins conjugated to ubiquitin, initially named heat-stable ATP-dependent proteolysis factor, were detected by Ciechanover and Hershko at a time, when scientists were perplexed by the paradox that proteins are turned over in an ATP-consuming manner after being synthesized by ATP consumption (Ciechanover et al., 1980; Hershko et al., 1980). Varshavsky and co-workers revealed that ubiquitin N-terminallyfused to galactose drastically reduced its half-live depending on the N-end rule, the N-terminal amino acid of galactose (Bachmair et al., 1986). The first poly-ubiquitylated substrates identified in cells were cyclins, cyclin-dependent kinase activators, and inhibitors regulating cell cycle progression (Kirschner, 1999). Also nascent polypeptides arising during protein translation are sources of proteasomal substrates, though their abundance might be less than originally assumed (Vabulas and Hartl, 2005). Poorly folded or misfolded nascent polypeptides may expose hydrophobic domains on the surface. If not instantaneously eliminated by proteasomal degradation, they are prone to nucleate toxic protein aggregations and the early onset of neurodegenerative diseases (Turner and Varshavsky, 2000; Navon and Goldberg, 2001; Medicherla and Goldberg, 2008).

## The 26S proteasome—the AAA-ATPase associated protease of eukaryotes

The 26S proteasomes exist in eukaryotic cells throughout the kingdom. They are composed of ~40 different protein subunits. Fourteen of these subunits are assembled in the proteolytic core particle (CP) which is composed of a stack of four seven-membered rings. Both outer rings contain seven alpha-subunits, both inner rings seven beta-subunits. The proteasome belongs to the class of self-compartmentalized threonine-proteases (Baumeister et al., 1998). The catalytic threonines conferred by three different beta-subunits are sequestered within the two inner beta-rings. Their substrate binding pockets have preferences for hydrophobic, basic, and acidic amino acids and are related to chymotrypsin-, trypsin-, and caspase-like peptide cleavage activities, respectively.

The outer alpha-rings form ante-chambers of the catalytic chambers enclosed between the two inner beta-rings (Tanaka, 2009). The central pores of the outer alpha rings are normally closed. N-terminal extensions of the alpha subunits occlude the central pores and restrict the diffusion of small chromogenic peptides used to assay proteolytic activities. Thus, free CP exhibits latent peptide cleavage activity (Groll et al., 1997; Orlowski and Wilk, 2000), at least under physiological potassium ion concentrations (Kisselev et al., 2002). Depending on the ions in the solution dynamic fluctuations between open and closed states of the CP exist as well as suggested by atomic force microscopy and NMR studies (Osmulski et al., 2009; Ruschak and Kay, 2012).

The conformational fluctuations of the central pores of the CP depend on sodium and potassium concentrations (Kohler et al., 2001; Osmulski et al., 2009). Detergents such as 0.02% SDS trigger the opening of the alpha-ring gates and allow free diffusion of chromogenic peptides into the CP. Not only detergents but also fatty acids, cardiolipin and polylysine open the alpha-ring gates and significantly stimulate peptide cleavage activity (Ichihara and Tanaka, 1989).

Thus, folded cellular proteins have restricted access to the proteolytic chamber to minimize nonspecific degradation. *In vitro*, natively disordered substrates can access the internal catalytic sites by threading their loose termini through the gates of the CP. Also loops lacking strong secondary structures can traverse the channel into the proteolytic cavity of the CP suggesting that intrinsically disordered protein (IDPs) domains trigger gate opening of the CP (Liu et al., 2003; Ben-Nissan and Sharon, 2014). To which extent IDPs are committed to proteasomal degradation remains to be examined, since IDPs might be shielded by chaperones belonging to the AAA-ATPase family and “nanny” proteins which insure their maturation into important regulatory and signaling proteins (Tsvetkov et al., 2009). Without protection proteins with IDPs might represent favored proteasomal substrates as long as they are not aggregated. Along these lines, disordered regions within regulatory and signaling proteins affect their half-life (Tsvetkov et al., 2012; van der Lee et al., 2014).

The gate opening of the CP is regulated by proteasome activators (PA), which relieve the autoinhibition of the CP by the N-terminal extensions of the alpha subunits. PA700, the regulatory complex (RP) of the eukaryotic proteasome, is the best-characterized PA and contains ~25 subunits. The RP binds to either one or both ends of the CP. The 240 kDa protein PA200/Blm10 is an alternative PA that is highly conserved from yeast to human. It stimulates the cleavage of small chromogenic peptides but does not contain AAA-ATPase activities required for polypeptide unfolding (Rechsteiner and Hill, 2005).

In contrast to these single protein PAs the RP is composed of ~25 different subunits which are assigned to two subcomplexes, the RP lid and base. Specifically, the RP base contains a hexameric ring of six subunits named Rpts in yeast or PSMCs in mammals that are members of the AAA-ATPase family (Glickman et al., 1998). The ATPase ring is adjacent to the CP alpha ring upon RP-CP binding (Baumeister et al., 1998). Newly advanced technologies using single particle cryo-EM provided detailed insight into the mechanism of how the ATPase ring is properly positioned for alpha ring opening to channel the translocation of unfolded substrates (Matyskiela and Martin, 2013; Unverdorben et al., 2014; Chen et al., 2016; Rodriguez-Aliaga et al., 2016).

## Substrate recognition by poly-ubiquitylation

Basically, in eukaryotic cells the poly-ubiquitin chain is the post-translational modification of a protein to be recognized as a potential substrate by the RP and to be recycled prior to degradation. Degrons are encoded in the amino acid sequence of the substrate which facilitate substrate processing. In the canonical sense, a chain of at least four isopeptide-conjugated ubiquitin molecules in combination with unstructured termini/loops within the substrate required to be recognized as degradation signal by the RP. Although all AAA-ATPases act on the protein substrate concurrently with the removal of the poly-ubiquitin chain, Rpt5, one of the Rpt ATPase subunits, was found to bind ubiquitin (Lam et al., 2002).

**Figure 1:**
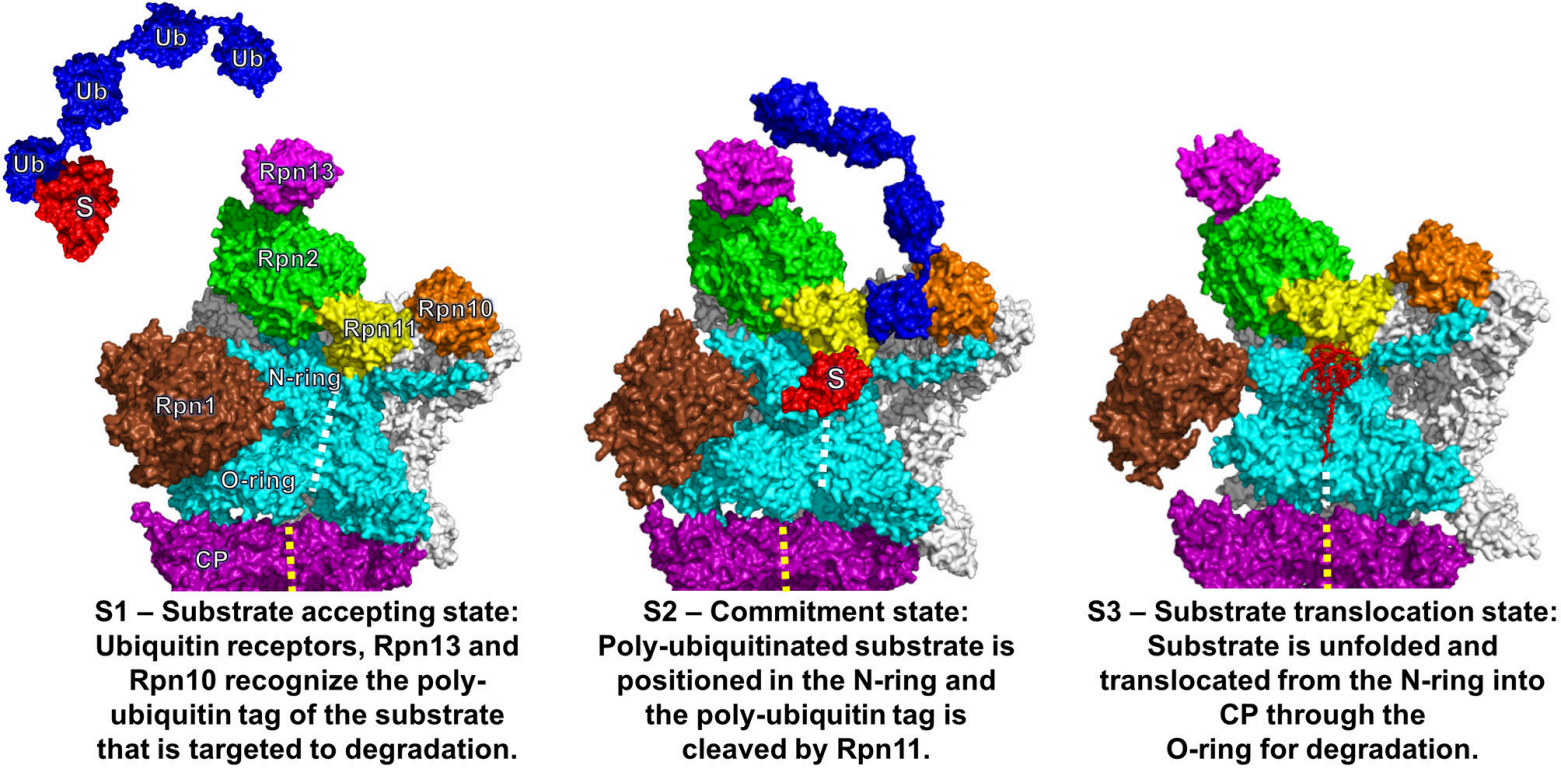
Three functional states of the eukaryotic proteasome. Conformational states of the eukaryotic proteasome, S1 (**Left**), S2 (**Middle**), and S3 (**Right**). The alpha ring of the catalytic core particle (CP) is colored in purple with full views of the regulatory particle (RP) for S1/S2 and longitudinal cross section for S3. The central channel through the CP and ATPase ring is indicated with yellow and white dashed lines, respectively. In the RP, the AAA-ATPase ring along with the N-terminal coiled-coils is colored in cyan. The non-ATPase RP subunits are colored in white except for Rpn1 (brown), Rpn2 (green), Rpn10 (orange), Rpn11 (yellow), and Rpn13 (magenta). In S1, a poly-ubiquitylated substrate (in red labeled with “S” attached to the tetra-ubiquitin chain in blue labeled with “Ub”) is recognized by the ubiquitin receptor Rpn13. Subsequently, the poly-ubiquitin chain is anchored to the ubiquitin receptor Rpn10 leading to substrate placement near the N-ring. In S2, the isopeptide bond between the substrate and poly-ubiquitin chain is cleaved by Rpn11 and the unfolding of the substrate is initiated. In S3, the unfolded substrate is translocated through the central pore of the AAA-ATPase ring into the central channel of the CP for degradation. The central pores of the AAA-ATPase O-ring and the CP are not aligned in S1 and S2 but are in S3. A 25° rotation of S1 to S2 facilitates the substrate placement into the N-ring and activates Rpn11. The figure was prepared using the PDB IDs: 4CR2, 4CR3, 4CR4, 1UBQ, 2ZNV, 2Z59, and 1UZX through PyMOL (Ver. 1.8.0.2) molecular graphics software (Schrodinger, LLC, New York).

To accommodate poly-ubiquitylated substrates, the proteasome shows a high degree of plasticity and versatility (Glickman and Raveh, 2005). Beyond shuttling ubiquitin receptors which transiently bind to ubiquitin-like domains on RP subunits, three RP subunit, namely Rpn1, Rpn10, and Rpn13 in yeast or PSMND2, PSMD4, and ADRM1 in mammals, serve as intrinsic docking sites for ubiquitin molecules (Finley, 2009; Rosenzweig et al., 2012; Shi et al., 2016). One major delivery site for poly-ubiquitin chains involves Rpn10 and Rpn13, the latter bound to Rpn2. The poly-ubiquitin chain is held between Rpn13 and Rpn10. The ubiquitin hydrolase Rpn11, a subunit of the RP lid and closely positioned to Rpn2 is responsible for the isopeptide-hydrolysis of the poly-ubiquitin chain. The polypeptide stripped off ubiquitin is adopted in an unfolded state by the adjacent AAA-ATPase ring. During the dynamic process of (i) substrate accepting, (ii) commitment, and (iii) translocation three hypothetic conformational states of the yeast proteasome were distinguished by single particle cryo EM analysis (Figure 1) (Lander et al., 2013; Unverdorben et al., 2014). The translocation state might be dissected into more intermediates, since human proteasomes exist in at least four states during substrate processing (Wehmer and Sakata, 2016).

The second delivery site for a poly-ubiquitin chain involves Rpn1 and Ubp6, named PSMD2 and USP14 in mammals. While the ubiquitin moieties are bound to Rpn1, the adjacent Ubp6 hydrolase trims super-numerous poly-ubiquitin chains. Again, the polypeptide cleaved off from the poly-ubiquitin chain is proposed to be furthered to the AAA-ATPase ring for unfolding and translocation into the CP (Shi et al., 2016), though Ubp6 is distant from the entrance pore of the AAA-ATPase ring. By trimming lengthy poly-ubiquitin chains the substrate can even escape final degradation, consistent with the finding that inhibition of Ubp6 stimulates protein degradation (Crosas et al., 2006).

A couple of additional ubiquitin receptors are known to ensnare Rpn1 and transiently deliver poly-ubiquitylated proteins to the RP (Rosenzweig et al., 2012). The remote binding of poly-ubiquitin chains most likely transmits allosteric conformational changes toward the coaxial CP alpha ring and the AAA-ATPase central pore to prepare the holo-enzyme for its commitment to protein degradation (Bech-Otschir et al., 2009; Peth et al., 2010).

Notably, poly-ubiquitin modifications are not compulsory for substrate degradation by proteasome holo-enzymes. One of the most prominent substrates that is degraded in an ATP-dependent matter without poly-ubiquitylation is ornithine decarboxylase (ODC) as elaborated by Coffino and co-workers (Erales and Coffino, 2014).

## Ancestors of AAA-ATPases in protein degradation

**Figure 2:**
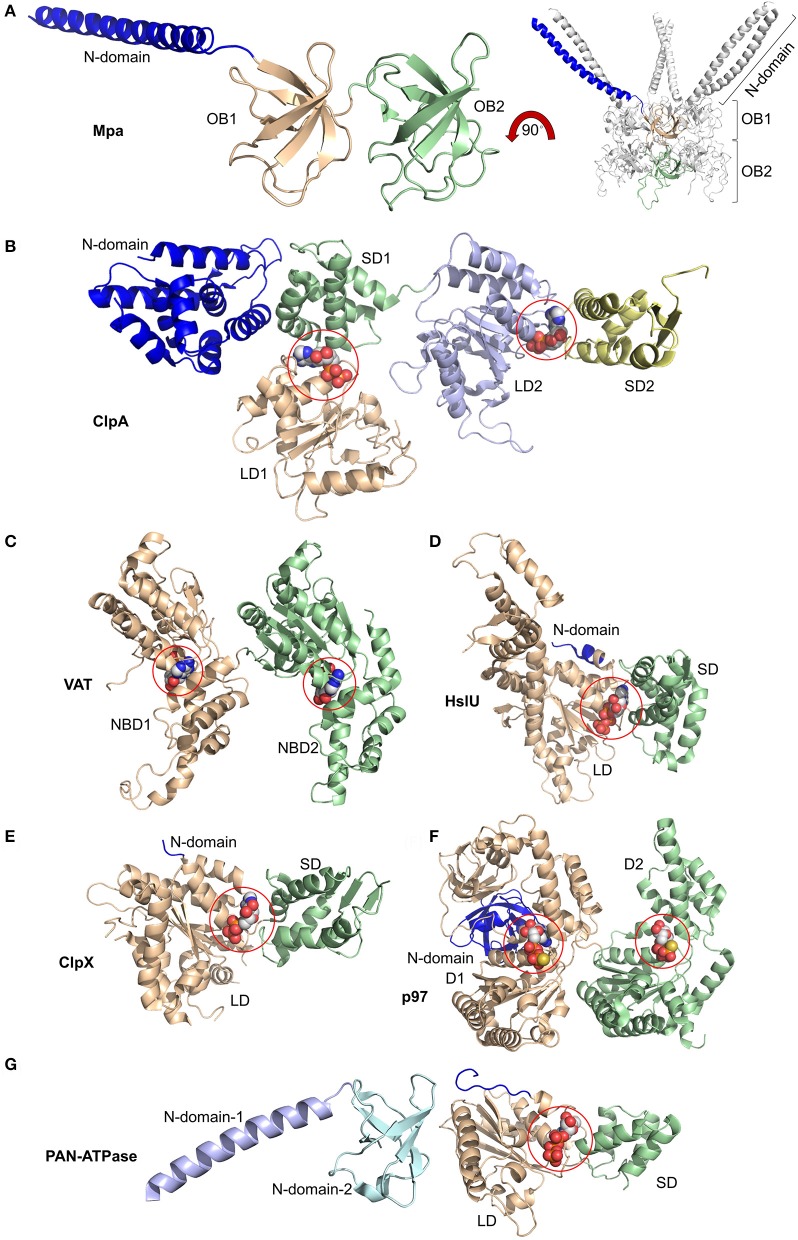
Domain organization of AAA-ATPases. **(A)** Magnified view of the monomer (left) and overall view of the oligomer (right) of Mpa containing two OB rings, OB1 and OB2, along with the N-terminal coiled-coils (blue). Magnified views of monomers bound to nucleotides highlighted by spheres of **(B)** ClpA with small and large domains SD1, SD2, LD1, and LD2 bound to ADP at the SD1/LD1 and SD2/LD2 interfaces; of **(C)** Valosin-containing protein-like ATPase (VAT) with nucleotide binding domains NBD1 and NBD2 bound to ATP; of **(D)** HslU with N-terminal (N), large (LD), and small (SD) domains and ATP; of **(E)** ClpX with N-terminal (N), large (LD), and small (SD) domains with ADP; of **(F)** p97/VCP/Cdc48 with N-terminal (N) and domain-1 (D1) and -2 (D2) bound to ATPγS; of **(G)** proteasome-activating nucleotidase (PAN) with N-domains 1 (from Gcn4) and 2 and large (LD) and small (SD) domains. Again, ATP is bound at the SD/LD interface. This figure was prepared based on the availability of structures in the protein data bank using the PDB IDs: 3M9D, 1KSF, 5VC7, 1DO0, 3HWS, 5C18, 2WG5, and 2WFW through PyMOL (Ver. 1.8.0.2) molecular graphics software (Schrodinger, LLC, New York).

Hexameric AAA-ATPase rings involved in ATP-dependent protein degradation exist in 26S proteasomes of eukaryotic cells and prokaryotic ancestors such as HsIU AAA-ATPase which is associated with HsIV protease composed of two homohexameric rings (Figure 2). In archaea proteasome-alike proteases composed of four heptameric rings are associated with VAT (Valosin-containing protein-like), the homolog of the ubiquitous AAA-ATPase Cdc48/p97, and PAN (Rockel et al., 2002; Benaroudj et al., 2003). In actinobacteria Mpa associates with the mycobacterial 20S proteasome, another evolutionary ancestor of eukaryotic proteasomes (Striebel et al., 2010). Hexameric AAA-ATPase rings also associate with prokaryotic AAA proteases such as ClpP, a serine protease composed of two heptameric rings. In these bacterial systems the AAA-ATPases are known as Clp ATPases (X, single ATPase ring; A, double ATPase ring) (Grimaud et al., 1998; Baker and Sauer, 2012).

Interfaces between the hexameric AAA-ATPase ring and the heptameric proteasome suggested a symmetry mismatch which precludes close complementary neighborhood and allow room for the dynamic changes underlying the mechanisms of protein translocation though the coaxial pores of the AAA-ATPase and the adjacent protease.

The architecture of prokaryotic AAA proteases is simple. The AAA-ATPase is a homohexamer. No RP equivalent is associated with the protease core. Bacterial proteases require no ubiquitin receptors, as ubiquitin signaling does not exist in prokaryotes (Jastrab and Darwin, 2015). Only in Mycobacteria tuberculosis, one of the world's deadliest pathogens, Pup, the prokaryotic ubiquitin-like protein, targets proteins by mono-pupylation for degradation. To recognize the Pup degradation tag, the N-terminal coiled coil regions of the AAA-ATPase Mpa homohexamer serve as template for the C-terminal half of Pup1 to fold into a helix (Wang et al., 2010). Beside the rare modification of pupylation, a variety of degrons exists which are encoded in the primary sequence and render a protein into a substrate. Due to their propensity for intrinsic disorder these degrons are prototype-patterns in protein degradation and not only recognized by prokaryotic but also by eukaryotic AAA proteases (Ravid and Hochstrasser, 2008; Varshavsky, 2011).

Most bacterial proteasomes are dodecamers of beta subunit ancestors. All other ancestor proteasomes with an exception of the bacterial species from *Rhodococcus* are composed of identical alpha and beta subunits. Their overall organization is similar. The alpha subunits are arranged in seven-membered outer rings, and the beta subunits in seven-membered inner rings, yielding a barrel-shaped particle with alpha7-beta7-beta7-alpha7 configuration as evidenced by the archaeal species from *Thermoplasma acidophilus* (Jastrab and Darwin, 2015). The opening by 23 Å of the alpha ring in bacterial proteasomes is wider than the opening by 13 Å in archaea, leading to a funnel through the center of the entire complex (Lowe et al., 1995).

In contrast to eukaryotic proteasomes where each of the seven distinct alpha subunits occupies a specific position to guarantee the closed-gate state, the N-terminal extensions of the identical alpha subunits of *Thermoplasma acidiphilum* proteasome are disordered and unable to lock the central pore (Lowe et al., 1995). Interestingly, the Mycobacterium proteasome has a closed gate, because the alpha-type subunits assume three different conformations. Three subunits form a rectangular shape (“L”), three form an extended linear shape (“E”), and one projects away to avoid a sterical clash (“V”) (Li et al., 2010).

Thus, in Mycobacterial and eukaryotic proteasomes the binding of AAA-ATPase rings facilitates the repositioning of the N-terminal extensions of the alpha subunits to open the central gates. The alpha ring gate of mycobacterial proteasomes can also be widened by Bpa, a just recently identified non-ATPase ring, suggesting that the AAA-ATPase activity is not required for alpha ring gating (Bolten et al., 2016).

**Figure 3:**
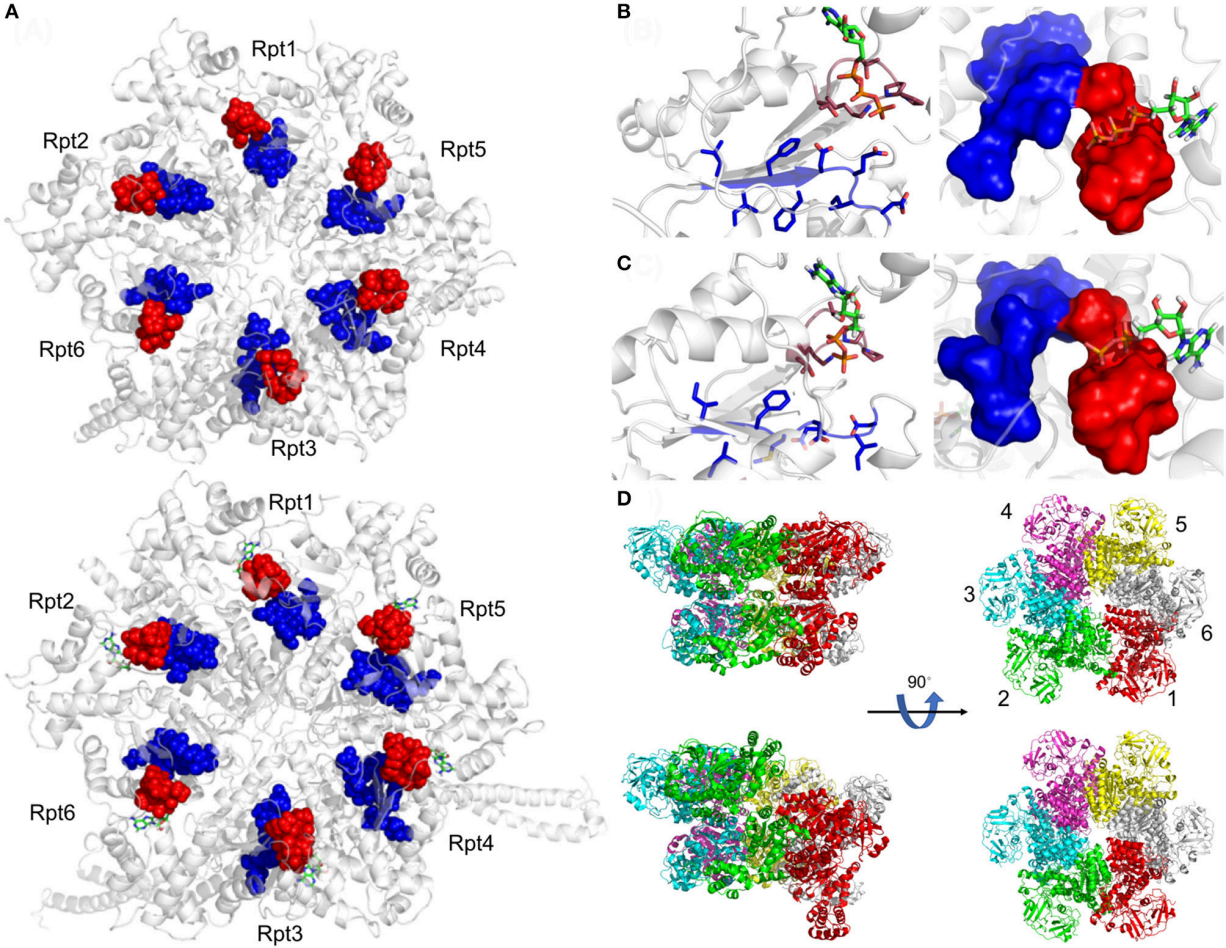
Active site organization of AAA-ATPase rings. **(A)** Bottom view of the proteasomal AAA-ATPase rings from yeast (upper panel) and human (lower panel). The Walker domain **A** is highlighted by red spheres and **B** by blue spheres. Magnified views of the Walker domains are shown for human AAA-ATPase bound to either ATP **(B)** or ADP **(C)** in two orientations. **(D)** Dynamics of Valosin-containing protein-like ATPase of *Thermoplasma acidophilum* (VAT) are visualized by conformational switches between the stacked and spiral (split-) ring versions. Side and top views of the AAA-ATPase subunit colored in red show movements out of the plane upon ATP hydrolysis aiding substrate translocation into the proteasome through its central pore. The split ring form (bottom left) undergoes a conformational change back into the stacked ring (top left), when ADP dissociates from the subunit and ATP binds back to allow the next round of hydrolysis. This figure was prepared using the PDB IDs: 4CR2, 5L4G, 5G4G, and 5G4F through PyMOL (Ver. 1.8.0.2) molecular graphics software (Schrodinger, LLC, New York).

Unlike the AAA-ATPase heterohexamer of the eukaryotic proteasome, the bacterial AAA-ATPase ring is a homohexamer (Striebel et al., 2009). Structurally, the prokaryotic AAA-ATPases resemble the eukaryotic counterparts. They all contain an alpha-helical domain close to the variable N-terminus followed by the oligonucleotide- and oligosaccharide binding domain (OB) and an AAA-ATPase domain, consisting of a RecA like subdomain and the α helical, C-terminal subdomain (Wendler et al., 2012). ATP binds to the Walker A motif between the two subdomains. Conserved loop residues line up the central pore of the AAA-ATPase ring which grip the unfolded protein substrate for translocation (Figure 3).

In PAN, which is an archaeal proteasomal AAA-ATPase ring, the six OB subdomains form the N-ring, while the N-terminal sequences adopt alpha-helical conformations and pair into three coiled coils. A conserved proline residue at the base of the N-terminal helix adopts a cis-conformation introducing a kink of the helix that allows coiled-coil formation with its neighbor subunit. To unfold and inject a protein the internal pore loops in the RecA like subdomain move the target protein toward its C-terminal end (Yu et al., 2010). PAN only transiently associates with 20S proteasomes from archaea (Barthelme and Sauer, 2012), unless a genetically engineered cystine bridge stabilizes the docking of the C-terminal HbYX motif in the alpha ring binding pocket of the 20S proteasome (Barthelme et al., 2014).

In general, Clp AAA-ATPases follow an ATP hydrolysis pattern different from eukaryotic AAA-ATPases. The ATP hydrolysis pattern is best studied in the homohexameric ClpX AAA-ATPase while velocity and processivity of most proteasomal AAA-ATPases still remain elusive (Lupas and Martin, 2002). The bacterial AAA-ATPase ClpX hydrolyzes ATP in a semi-stochastic way with a hydrolysis rate of ~100–500 ATP molecules per minute in the absence of substrate. In association with a protease substrates are degraded with high velocity but low processivity slipping back and forth, once the AAA-ATPase encounters a folded domain (Aubin-Tam et al., 2011; Maillard et al., 2011; Nager et al., 2011; Baytshtok et al., 2015; Iosefson et al., 2015; Rodriguez-Aliaga et al., 2016).

## The AAA-ATPases of the eukaryotic proteasome

In contrast to the prokaryotic systems, the AAA-ATPase of the eukaryotic proteasome is a heterohexamer suggesting specialization among the six different ATPase subunits (Rubin et al., 1998). The six ATPase subunits arrange in a particular order: Rpt1-Rpt2-Rpt6-Rpt3-Rpt4-Rpt5 (Tomko and Hochstrasser, 2011) (Figure 3). The N-terminal domains form coiled-coils, as Rpt2, Rpt3, and Rpt5 contain a conserved proline residue at the base of the helix that build coiled-coils with significant differences in their length and breaks of the symmetry.

**Figure 4:**
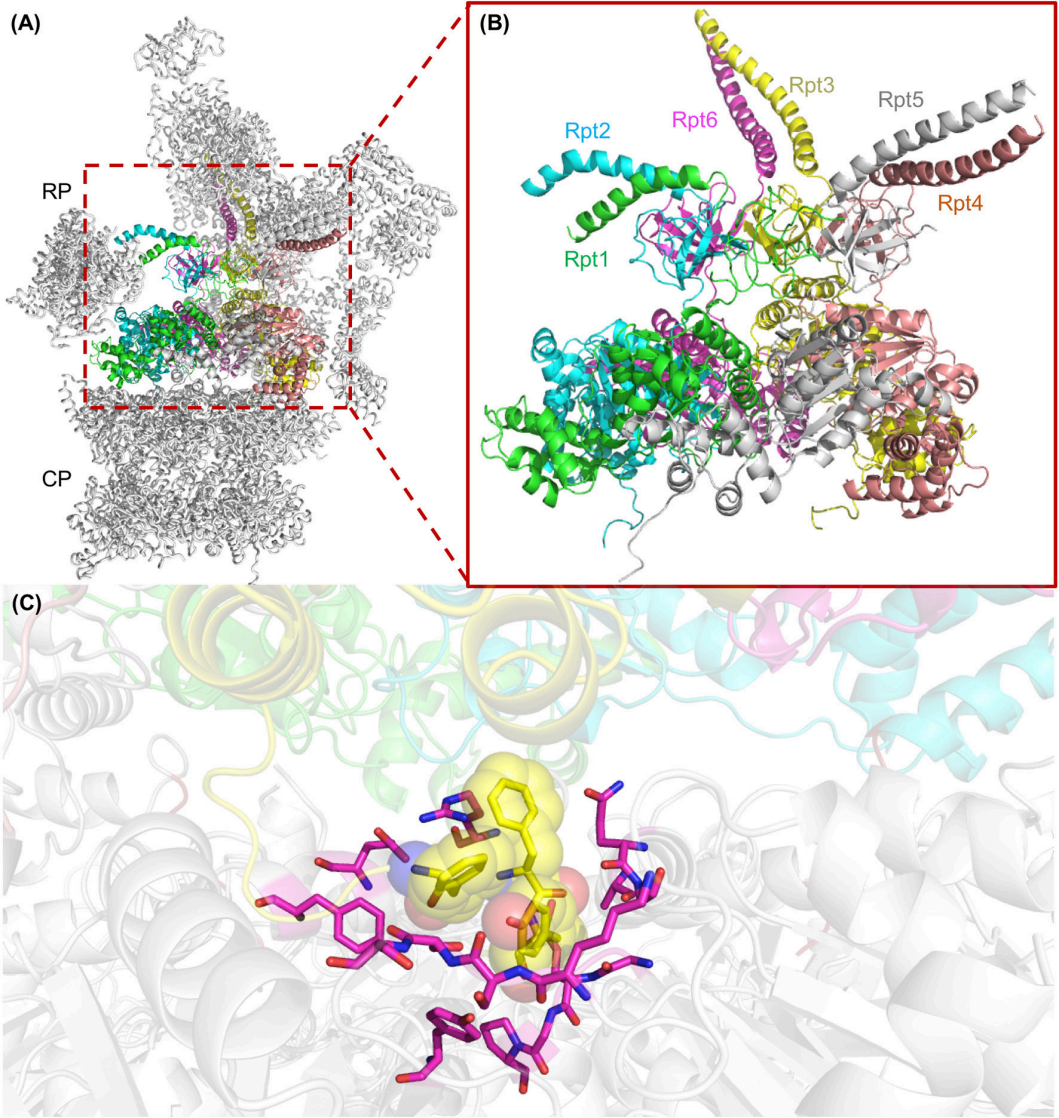
The AAA+ ATPase ring of the yeast 26S proteasome. **(A)** Structure of an assembled yeast proteasome showing half of the catalytic core particle (CP) attached to the regulatory particle (RP). The AAA-ATPase ring is highlighted in colors. **(B)** A magnified view of the AAA-ATPase ring containing the subunits Rpt1 to Rpt6. The N-terminal coiled-coils are formed by Rpt1 and 2, Rpt4 and 5, and Rpt3 and 6. **(C)** The interface between the ATPase ring and the CP is shown with the HbYX motif at the C-terminus of Rpt3 (highlighted as yellow spheres) digging into the alpha subunit binding pocket of the CP. Residues of CP alpha subunits that line the binding pocket of the HbYX motif are highlighted in magenta. This figure was prepared using the PDB ID: 4CR2 through PyMOL (Ver. 1.8.0.2) molecular graphics software (Schrodinger, LLC, New York).

The binding of the proteasomal AAA-ATPase ring to the alpha ring of the proteasome requires the highly conserved penultimate tyrosine residue within the C-terminal HbYX (hydrophobic-tyrosine-any amino acid) motif (Smith et al., 2007; Rabl et al., 2008). Upon ATP binding the subunits with HbYX motifs bind to inter-pockets between two alpha subunits of the CP like a “key in a lock” (Figure 4). With bacterial AAA-ATPases consisting of homohexamers six identical HbYX motifs can interact with seven alpha subunit pockets, stabilizing the interactions of the ATPase AAA protease complex (Jastrab and Darwin, 2015).

**Figure 5:**
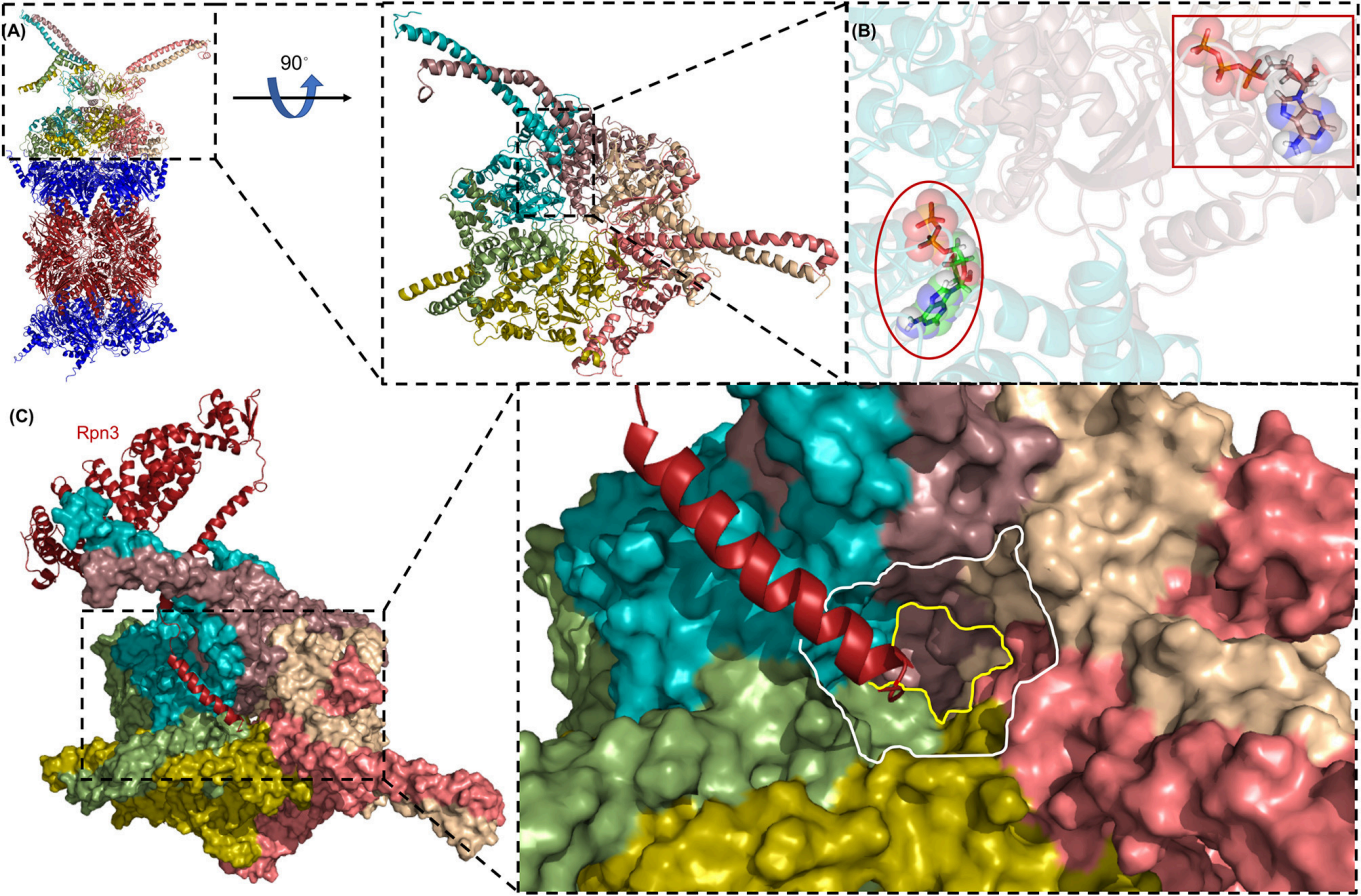
The AAA-ATPase ring of the human proteasome. **(A)** The AAA-ATPase is located on the alpha ring of the CP. The catalytic beta-subunits are colored in red, the alpha-subunits in blue. A magnified view of the AAA-ATPase ring is shown on the right. Coiled-coils of N-terminal regions reach out to other RP subunits. **(B)** The AAA-ATPase subunit colored in cyan is bound to ADP (red ellipse), while the other five AAA-ATPase subunits are bound to ATP (red box). **(C)** Rpn3 acts as sensor to induce conformational changes in the RP upon substrate docking into the ATPase ring (shown as a surface diagram). The C-terminus of Rpn3 colored in red is close to the pore of the N-ring (white line) and the O-ring (yellow line). This figure was prepared using the PDB ID: 5L4G through PyMOL (Ver. 1.8.0.2) molecular graphics software (Schrodinger, LLC, New York).

Though four out of six proteasomal AAA-ATPases, namely Rpt1, 2, 3, and 5, have HbYX motifs, gate opening could be induced by C-terminal peptides of Rpt2 and Rpt5 suggesting that the hexameric ATPase ring is mainly anchored by two contact sites to the heptameric ring of the alpha subunits (Smith et al., 2007). In the proteasome purified from yeast, the C-terminal HbYX motifs of Rpt2, Rpt3, and Rpt5 turned out to bind to the inter-pockets between alpha 3–4, 1–2, and 5–6, respectively. A rotation in the alpha subunits and displacement of a reverse-turn loop occluding the central pore are induced, so that the open gate conformation is stabilized within the holo-enzyme and substrate entry is facilitated (Rabl et al., 2008; Park et al., 2013) (Figure 5).

The loops of the ATPase subunit lining the central pore of the hexamer are suspected to contact the substrate to be unfolded. ATP hydrolysis triggers conformational changes of individual ATPase subunits that exert a pulling force to unfold and translocate the substrate through the narrow central pore of the CP alpha ring which is consecutively widened enough to accommodate an unfolded polypeptide chain. The hydrolysis rate of proteasomal AAA-ATPases is ~30–50 molecules of ATP per minute which is considerably slower than the rate of ClpX AAA-ATPases (Hoffman and Rechsteiner, 1996; Kraut et al., 2012; Kim et al., 2015). The slow velocity allows more processivity during substrate degradation, that the machinery does not stall but rather drives through without slipping, when it approaches a folded domain (Smith et al., 2011; Kim et al., 2015).

In eukaryotic proteasomes the substrate polypeptide is engaged with the unfolding activity of the AAA-ATPase ring concurrently with the removal of the tetra-ubiquitin chain but the recognition of the poly-ubiquitin chain is not sufficient for degradation. The proteolytic engagement requires an unstructured initiation site which reaches through the OB-domain containing N-ring to the AAA-pore (Prakash et al., 2004).

The site of the poly-ubiquitin chain to be cleaved off must be approximately thirty amino acids away where the ubiquitin isopeptidase activity of Rpn11 is located. The length of approximately thirty amino acids is also required for proteasomal model substrates with accessible termini, that are degraded *in vitro* by proteasomes independently of ubiquitination (Kraut et al., 2007; Takeuchi et al., 2007). In some instances, one or two ubiquitin molecules are already sufficient for signaling degradation suggesting that the tetra-ubiquitin chain is not necessarily a switch-on for degradation. The question is what could be the molecular ruler beside the poly-ubiquitin chain on which a protein is recognized as proteasomal substrate. The susceptibility of the unstructured regions of the substrate to unfolding determines the efficacy of degradation rather than the anchoring of ubiquitin to the proteasome (Prakash et al., 2004). Also the size of the protein seems to determine the pathway of degradation in favor of mono- over poly-ubiquitylation (Shabek et al., 2012). The accessibility of lengthy poly-ubiquitin chains to VAT/Cdc48, an abundant ubiquitous AAA-ATPase transiently interacting with archaeal proteasomes, also influences the fate of a proteasomal substrate, as Cdc48 facilitates the extraction of protein substrates stuck into membranes and protein aggregates (Godderz et al., 2015).

## Newest insights into the detailed mechanism of AAA-ATPases

According to current models of AAA-ATPases individual subunits are in different stages of the ATPase cycle. Prokaryotic AAA-ATPases such as ClpX hydrolyze ATP in a semi-stoichastic manner, whereas eukaryotic AAA-ATPases of the proteasome are suggested to hydrolyze ATP in an ordered and sequential cycle by binding ATP molecules to the ortho position (direct neighboring subunit) of the hydrolyzed ATP molecule. Allostery between eukaryotic AAA-ATPase subunits is mediated by trans-arginine-fingers which are lacking in ClpX reflecting structural differences with regard to ATP hydrolysis and potentially resulting in distinct strategies for protein unfolding (Kim et al., 2015). ATP binding and hydrolysis induce coordinated conformational changes (Smith et al., 2011; Stinson et al., 2013). With saturating ATP concentration, all six Rpts adopt a staircase arrangement, with Rpt3 at the highest step and Rpt2 at the lowest step relative to the CP, whereas the C-terminal domains are positioned in a plane above the CP (Lander et al., 2012). Engaged with a substrate the staircase arrangement is no more present (Matyskiela and Martin, 2013).

Subunit staggering and staircase arrangements are not due to the asymmetry of the heterohexameric ATPase ring of RP. It has been observed for prokaryotic homohexameric ATPases as well (Thomsen and Berger, 2009).

Could the stair case configuration be static and represent the optimal acceptor state for incoming polypeptides that have to be accommodated from different sites above the central entry pore? Ubiquitin-hydrolyzing activities by Ubp6 and Rpn11 and their corresponding ubiquitin receptor sites are asymmetrically positioned in the RP and hover above the substrate entry port of the Rpt ATPase ring. Substrate or ATP binding may swing the active site of Rpn11 toward the central pore of the AAA-ATPase from a discontinuous conformation to a position in which the AAA-ATPase pore is properly aligned with the alpha ring gate of the CP (Matyskiela and Martin, 2013).

The archaeal VAT ATPase, the archaeal counterpart of Cdc48/p97, showed a staircase arrangement of the homohexameric ring, when at least one subunit was bound to ADP (Huang et al., 2016). Mutations in critical tyrosines of the VAT-pore loops cause defects in protein unfolding and translocation (Gerega et al., 2005). Snapshots obtained by cryo EM and NMR studies revealed that the movement between stacked and split-ring structures for VAT suggests repeated cycles of ATP binding and hydrolysis by setting the central pore on different heights to generate the pulling force on the substrate. They reflect substrate-AAA pore loop contacts with the translocation channel into the proteasome (Figure 3D). Transient intermediates of substrate translocation through VAT ATPase were captured by cryo EM. Substrate binding breaks the six-fold symmetry, allowing five of the six VAT subunits to constrict into a tight helix that grips an ~80 Å stretch of unfolded protein. The structure suggests a processive hand-over-hand unfolding mechanism, where each VAT subunit releases the substrate in turn before re-engaging further along the target protein, thereby unfolding it (Ripstein et al., 2017).

All mechanistic studies on AAA-ATPase before occurred on idle hexamers with no unfolded peptide in the process of translocation (Ripstein et al., 2017). How many of the six subunits of the hexamer are actually loaded with nucleotides, is not definitively determined, unless we assume that the subunits were oversaturated with either non-hydrolyzable analogs of ATP and completely bound to ADP. Negative allostery might be possible when ATP binding to one site prevents nucleotide binding to another site. Furthermore, it is unclear whether the six subunits of the ATPase have hydrolyzed ATP in a random, sequential or concerted manner.

Electron cryo-tomography in cells also revealed asymmetrically twisted Rpt ATPase rings in 26S proteasomes which were assigned to enzymes engaged in degradation compared with idle enzymes in the ground state after ATP hydrolysis. The AAA pore loops are aligned in a spiral plane in the ground state and in a nearly planar configuration in the engaged state (Matyskiela and Martin, 2013; Unverdorben et al., 2014).

When active site mutants in Rpt subunits were compared, the most severe effects on protein degradation were observed for mutations in Rpt subunits within pore loops closest to the substrate entry point in the OB-containing N-ring pointing to the hot spot, the “commitment step” for final degradation (Erales et al., 2012; Beckwith et al., 2013).

Recent advances in dual-laser optical trapping technologies on single molecules allowed testing the existing models of protein unfolding and degradation. Sophisticated reporter substrates such as ssrA-degron-(unfolded Titin)_4_ were engineered to measure the mechanical forces that apply on these substrates during translocation (Maillard et al., 2011; Sen et al., 2013; Cordova et al., 2014). Bacterial ClpP protease bound to either double ring AAA-ATPase ClpA or single ring ATPase ClpX were compared for the translocation capacity of the reporter substrate. It was substantially faster degraded but slower translocated by the protease with the ClpA double ring compared with the ClpX single ring (Olivares et al., 2016). The fundamental translocation step is independent of double or single ring architecture supporting the conclusion that constrains imposed by the nucleotide state determine the size of a single power stroke (Glynn et al., 2009; Stinson et al., 2013). Similar settings in a dual optical tweezer assay using a GFP-labeled variant of ssrA-degron-(unfolded Titin)_4_ allowed further characterization of the mechanochemical cycle of ClpXP. The AAA-ATPase motor is cycling through two phases. In the dwell phase ClpXP does not move its substrate. In the burst phase CplXP pushes the substrate in increments of few nanometers, resulting in a near simultaneous ATP-driven conformational change of single ATPase subunits, thereby propelling the substrate via individual power strokes (Aubin-Tam et al., 2011). ADP release and ATP binding occurred in the dwell phase, whereas ATP hydrolysis and phosphate release happened in the burst phase. Conformational re-settings of the pore loops appear to determine the time for ADP release from individual ATPase subunits (Rodriguez-Aliaga et al., 2016).

Recent single particle cryo-EM analysis of human 26S proteasomes to near-atomic resolution provided complementary information about the substrate-unfolding AAA-ATPase channel in its nucleotide-bound state (Chen et al., 2016) (Figure 5). The AAA pore is shaped by inward facing pore loops, which are arranged in two parallel helixes, one is populated with hydrophobic and the other with charged amino acid residues. The interior of the AAA channel is negatively charged, the interior of the OB channel positively charged. Both parts of the channel are enriched by crucial tyrosine residues, which feature the conserved hydrophobic Tyr/Phe-Val/Leu/Ile-Gly pattern. The resolution of this critical region of the AAA-ATPase allowed the differentiation of four proteasome configurations. Six ATP molecules were tentatively modeled into the binding pockets, because the nucleotide state could not be determined for each Rpt subunit due to the averaging of single particle images. Surprisingly, the alpha rings of the CP were closed in three out of four conformations. The Rpt subunits seem to be in direct contact with the alpha ring of the CP by anchoring the HbYX motif of Rpt5 but not of Rpt2 into the respective inter-pocket of the CP alpha ring. Movements of the Rpt subunits on the alpha ring eventually facilitate the reach out of the HbYX motifs to Rpt1, 2, and 6 to their nearest inter-pockets, until remaining gate-blocking C-terminal tails align along the center axis of the pore. Taken together, the opening is primed through a series of coordinated, stepwise remodeling events including the RP lid swinging in the appropriate position above the axial channel (Chen et al., 2016). The configuration of the ground state with the closed CP gate was consistent with recent high-resolution cryo-EM structures (Huang et al., 2016; Schweitzer et al., 2016). Rpt6 is structurally distinct from the other five Rpt subunits, most notably in its pore loop region. Moreover, the C terminus of Rpn3 was found to protrude into the ATPase ring and proposed to trigger conformational changes to the AAA-ATPase ring (Figure 5). Rpn1 and Rpn2, the largest proteasome subunits, are linked by an extended alpha helix suggesting coordinated co-operations between the RP ATPases and non-ATPases to orchestrate substrate recognition, unfolding and translocation (Schweitzer et al., 2016).

## Escape mechanisms of AAA proteases

The proteasome is committed to operate processively on a substrate and determines the substrate's fate (Lee et al., 2001). However, successful initiation of substrate translocation, presumably by the synergistic interaction between the AAA pore loops and the translocation channel into the CP, does not guarantee the execution of proteolysis, when pore loop interactions with the gripped substrates are lost, especially when slippery elements of low complexity or intrinsically disordered sequences are positioned adjacent to folded domains. Especially, repetitive sequences of glycine-alanine residues resulted in the blockage of degradation, because the AAA-ATPase seems to slip on the repetitive sequences without being able to grasp the polypeptide (Levitskaya et al., 1995; Zhang and Coffino, 2004). The preferences of the AAA-ATPases for specific sequences seem to provide an additional component to the degradation code and may fine-tune the half-lives of cellular proteins. Clusters of glutamate repeats inhibited degradation of the protein (Fishbain et al., 2015), possibly by being repulsed by negatively charged amino acid residues in the AAA-pore (Chen et al., 2016). Ubiquitin-associated domains (UBA) protect against proteasomal degradation which is detrimental for shuttling ubiquitin receptors such as Rad23 and Dsk2 which deliver poly-ubiquitylated substrates to the proteasome without being sacrificed. Insertion of an UBA domain near an intrinsically disordered region stabilizes the protein (Heessen et al., 2005; Heinen et al., 2011).

Tetra-ubiquitin can also be covalently linked to a subunit of a protein complex to be targeted to the proteasomes without being degraded, because the subunit is sufficiently folded and not extracted by the Rpt ATPases. Instead, the neighboring subunit having an intrinsically disordered domain is degraded (Prakash et al., 2004). Also the other way around is known that a ubiquitinated subunit of a complex is degraded, while the neighboring subunit remains intact (Hochstrasser and Varshavsky, 1990; Johnson et al., 1990; Verma et al., 2001). Thus, the Rpt AAA-ATPases seem to favor the substrate with the easiest accessible termini and the most likely initiation site, an unstructured region penetrating to the ATPase pore loops. Unstructured regions such as the 37 amino acid long C-terminal tail of ODC, bind so tightly to the AAA-ATPase that poly-ubiquitination is not required for degradation as known for other degrons in the bacterial and archaeal system (Erales and Coffino, 2014). *In vitro*, proteins with largely unstructured regions such as NQO1 are even being degraded by the CP without the aid of Rpt AAA-ATPases, but this mechanism is yet to be verified *in vivo* (Moscovitz et al., 2012).

The RP base complex harboring the Rpt AAA-ATPases was also shown to exhibit foldase activity of AAA-ATPase chaperones. Denatured citrate synthase without ubiquitin modification was refolded and reactivated by Rpt ATPase without being degraded by proteasomes (Braun et al., 1999).

Finally, proteasomal AAA-ATPases have also been propsed to be involved in non-proteolytic re-folding processes such as nucleotide excision repair (Gillette et al., 2001; Gonzalez et al., 2002). DNA microarrays revealed RP subunits but no CP subunits to be associated with chromosomal DNA. However, the experimental conditions under which chromatin immunoprecipitation assays are performed may weaken the interaction between RP and CP resulting in the dissociation of the CP from the RP.

## Outlook

Different—and sometimes incompatible—models based on NMR, X-ray, and cryo-EM structure analysis are available to visualize important steps in protein substrate unfolding and translocation through AAA-ATPases which are associated with proteasomes and proteasome-like proteases. The usage of optical tweezers and fluorescence microscopy on single molecules allowed the first comprehensive mechanochemical characterization of a bacterial AAA-ATPase. Its motor power reconciles the product of generated force and translocation velocity. This novel approach is expected to add detailed pictures of how the chemical transitions in the ATPase cycle of an AAA-ATPase are coupled to the dwell and burst phases of the motor between its grip on the substrate and its pulling frequency. Future studies based on this technology will reveal whether related AAA-ATPases, including the eukaryotic 26S proteasome, may use similar mechanisms for ATP-dependent substrate unfolding and translocation.

## Author contributions

CE is the corresponding author, wrote the first draft of the manuscript and approved the final version for publication. RY prepared the Figures and Figure legends. PW has substantial, direct and intellectual contribution to the work, and approved it for publication.

### Conflict of interest statement

The authors declare that the research was conducted in the absence of any commercial or financial relationships that could be construed as a potential conflict of interest.

## References

[B1] Aubin-Tam M. E. Olivares A. O. Sauer R. T. Baker T. A. Lang M. J. (2011). Single-molecule protein unfolding and translocation by an ATP-fueled proteolytic machine. Cell 145, 257–267. 10.1016/j.cell.2011.03.036 21496645 PMC3108460

[B2] Bachmair A. Finley D. Varshavsky A. (1986). *In vivo* half-life of a protein is a function of its amino-terminal residue. Science 234, 179–186. 10.1126/science.3018930 3018930

[B3] Baker T. A. Sauer R. T. (2012). ClpXP, an ATP-powered unfolding and protein-degradation machine. Biochim. Biophys. Acta 1823, 15–28. 10.1016/j.bbamcr.2011.06.007 21736903 PMC3209554

[B4] Barthelme D. Chen J. Z. Grabenstatter J. Baker T. A. Sauer R. T. (2014). Architecture and assembly of the archaeal Cdc48•20*S* proteasome. Proc. Natl. Acad. Sci. U.S.A. 111, E1687–E1694. 10.1073/pnas.1404823111 24711419 PMC4035981

[B5] Barthelme D. Sauer R. T. (2012). Identification of the Cdc48•20S proteasome as an ancient AAA+ proteolytic machine. Science 337, 843–846. 10.1126/science.1224352 22837385 PMC3923512

[B6] Baumeister W. Walz J. Zühl F. Seemüller E. (1998). The proteasome: paradigm of a self-compartmentalizing protease. Cell 92, 367–380. 10.1016/S0092-8674(00)80929-0 9476896

[B7] Baytshtok V. Baker T. A. Sauer R. T. (2015). Assaying the kinetics of protein denaturation catalyzed by AAA+ unfolding machines and proteases. Proc. Natl. Acad. Sci. U.S.A. 112, 5377–5382. 10.1073/pnas.1505881112 25870262 PMC4418879

[B8] Bech-Otschir D. Helfrich A. Enenkel C. Consiglieri G. Seeger M. Holzhätter H. G.. (2009). Polyubiquitin substrates allosterically activate their own degradation by the 26S proteasome. Nat. Struct. Mol. Biol. 16, 219–225. 10.1038/nsmb.1547 19169257

[B9] Beckwith R. Estrin E. Worden E. J. Martin A. (2013). Reconstitution of the 26S proteasome reveals functional asymmetries in its AAA+ unfoldase. Nat. Struct. Mol. Biol. 20, 1164–1172. 10.1038/nsmb.2659 24013205 PMC3869383

[B10] Benaroudj N. Zwickl P. Seemäller E. Baumeister W. Goldberg A. L. (2003). ATP hydrolysis by the proteasome regulatory complex PAN serves multiple functions in protein degradation. Mol. Cell 11, 69–78. 10.1016/S1097-2765(02)00775-X 12535522

[B11] Ben-Nissan G. Sharon M. (2014). Regulating the 20S proteasome ubiquitin-independent degradation pathway. Biomolecules 4, 862–884. 10.3390/biom4030862 25250704 PMC4192676

[B12] Bolten M. Delley C. L. Leibundgut M. Boehringer D. Ban N. Weber-Ban E. (2016). Structural analysis of the bacterial proteasome activator Bpa in complex with the 20S proteasome. Structure 24, 2138–2151. 10.1016/j.str.2016.10.008 27839949

[B13] Braun B. C. Glickman M. Kraft R. Dahlmann B. Kloetzel P. M. Finley D.. (1999). The base of the proteasome regulatory particle exhibits chaperone-like activity. Nat. Cell Biol. 1, 221–226. 10.1038/12043 10559920

[B14] Chen S. Wu J. Lu Y. Ma Y. B. Lee B. H. Yu Z.. (2016). Structural basis for dynamic regulation of the human 26S proteasome. Proc. Natl. Acad. Sci. U.S.A. 113, 12991–12996. 10.1073/pnas.1614614113 27791164 PMC5135334

[B15] Ciechanover A. Brundin P. (2003). The ubiquitin proteasome system in neurodegenerative diseases: sometimes the chicken, sometimes the egg. Neuron 40, 427–446. 10.1016/S0896-6273(03)00606-8 14556719

[B16] Ciechanover A. Heller H. Elias S. Haas A. L. Hershko A. (1980). ATP-dependent conjugation of reticulocyte proteins with the polypeptide required for protein degradation. Proc. Natl. Acad. Sci. U.S.A. 77, 1365–1368. 10.1073/pnas.77.3.1365 6769112 PMC348495

[B17] Cordova J. C. Olivares A. O. Shin Y. Stinson B. M. Calmat S. Schmitz K. R.. (2014). Stochastic but highly coordinated protein unfolding and translocation by the ClpXP proteolytic machine. Cell 158, 647–658. 10.1016/j.cell.2014.05.043 25083874 PMC4134808

[B18] Crosas B. Hanna J. Kirkpatrick D. S. Zhang D. P. Tone Y. Hathaway N. A.. (2006). Ubiquitin chains are remodeled at the proteasome by opposing ubiquitin ligase and deubiquitinating activities. Cell 127, 1401–1413. 10.1016/j.cell.2006.09.051 17190603

[B19] Erales J. Coffino P. (2014). Ubiquitin-independent proteasomal degradation. Biochim. Biophys. Acta 1843, 216–221. 10.1016/j.bbamcr.2013.05.008 23684952 PMC3770795

[B20] Erales J. Hoyt M. A. Troll F. Coffino P. (2012). Functional asymmetries of proteasome translocase pore. J. Biol. Chem. 287, 18535–18543. 10.1074/jbc.M112.357327 22493437 PMC3365715

[B21] Finley D. (2009). Recognition and processing of ubiquitin-protein conjugates by the proteasome. Annu. Rev. Biochem. 78, 477–513. 10.1146/annurev.biochem.78.081507.101607 19489727 PMC3431160

[B22] Fishbain S. Inobe T. Israeli E. Chavali S. Yu H. Kago G.. (2015). Sequence composition of disordered regions fine-tunes protein half-life. Nat. Struct. Mol. Biol. 22, 214–221. 10.1038/nsmb.2958 25643324 PMC4351145

[B23] Gerega A. Rockel B. Peters J. Tamura T. Baumeister W. Zwickl P. (2005). VAT, the thermoplasma homolog of mammalian p97/VCP, is an N domain-regulated protein unfoldase. J. Biol. Chem. 280, 42856–42862. 10.1074/jbc.M510592200 16236712

[B24] Gillette T. G. Huang W. Russell S. J. Reed S. H. Johnston S. A. Friedberg E. C. (2001). The 19S complex of the proteasome regulates nucleotide excision repair in yeast. Genes Dev. 15, 1528–1539. 10.1101/gad.869601 11410533 PMC312714

[B25] Glickman M. H. Raveh D. (2005). Proteasome plasticity. FEBS Lett. 579, 3214–3223. 10.1016/j.febslet.2005.04.048 15890341

[B26] Glickman M. H. Rubin D. M. Fried V. A. Finley D. (1998). The regulatory particle of the *Saccharomyces cerevisiae* proteasome. Mol. Cell. Biol. 18, 3149–3162. 10.1128/MCB.18.6.3149 9584156 PMC108897

[B27] Glynn S. E. Martin A. Nager A. R. Baker T. A. Sauer R. T. (2009). Structures of asymmetric ClpX hexamers reveal nucleotide-dependent motions in a AAA+ protein-unfolding machine. Cell 139, 744–756. 10.1016/j.cell.2009.09.034 19914167 PMC2778613

[B28] Godderz D. Heinen C. Marchese F. P. Kurz T. Acs K. Dantuma N. P. (2015). Cdc48-independent proteasomal degradation coincides with a reduced need for ubiquitylation. Sci. Rep. 5:7615. 10.1038/srep07615 25556859 PMC5154593

[B29] Goldberg A. L. (2003). Protein degradation and protection against misfolded or damaged proteins. Nature 426, 895–899. 10.1038/nature02263 14685250

[B30] Gonzalez F. Delahodde A. Kodadek T. Johnston S. A. (2002). Recruitment of a 19S proteasome subcomplex to an activated promoter. Science 296, 548–550. 10.1126/science.1069490 11964484

[B31] Grimaud R. Kessel M. Beuron F. Steven A. C. Maurizi M. R. (1998). Enzymatic and structural similarities between the *Escherichia coli* ATP-dependent proteases, ClpXP and ClpAP. J. Biol. Chem. 273, 12476–12481. 10.1074/jbc.273.20.12476 9575205

[B32] Groll M. Ditzel L. Lowe J. Stock D. Bochtler M. Bartunik H. D.. (1997). Structure of 20S proteasome from yeast at 2.4 A resolution. Nature 386, 463–471. 10.1038/386463a0 9087403

[B33] Heessen S. Masucci M. G. Dantuma N. P. (2005). The UBA2 domain functions as an intrinsic stabilization signal that protects Rad23 from proteasomal degradation. Mol. Cell 18, 225–235. 10.1016/j.molcel.2005.03.015 15837425

[B34] Heinen C. Acs K. Hoogstraten D. Dantuma N. P. (2011). C-terminal UBA domains protect ubiquitin receptors by preventing initiation of protein degradation. Nat. Commun. 2, 191. 10.1038/ncomms1179 21304520 PMC3105319

[B35] Hershko A. Ciechanover A. Heller H. Haas A. L. Rose I. A. (1980). Proposed role of ATP in protein breakdown: conjugation of protein with multiple chains of the polypeptide of ATP-dependent proteolysis. Proc. Natl. Acad. Sci. U.S.A. 77, 1783–1786. 10.1073/pnas.77.4.1783 6990414 PMC348591

[B36] Hochstrasser M. Varshavsky A. (1990). *In vivo* degradation of a transcriptional regulator: the yeast alpha 2 repressor. Cell 61, 697–708. 10.1016/0092-8674(90)90481-S 2111732

[B37] Hoffman L. Rechsteiner M. (1996). Nucleotidase activities of the 26 S proteasome and its regulatory complex. J. Biol. Chem. 271, 32538–32545. 10.1074/jbc.271.51.32538 8955078

[B38] Huang R. Ripstein Z. A. Augustyniak R. Lazniewski M. Ginalski K. Kay L. E.. (2016). Unfolding the mechanism of the AAA+ unfoldase VAT by a combined cryo-EM, solution NMR study. Proc. Natl. Acad. Sci. U.S.A. 113, E4190–E4199. 10.1073/pnas.1603980113 27402735 PMC4961139

[B39] Huang X. Luan B. Wu J. Shi Y. (2016). An atomic structure of the human 26S proteasome. Nat. Struct. Mol. Biol. 23, 778–785. 10.1038/nsmb.3273 27428775

[B40] Ichihara K. Tanaka C. (1989). Progesterone metabolism in the gastric mucosa microsomes of guinea pig. J. Steroid Biochem. 32, 835–840. 10.1016/0022-4731(89)90460-3 2547113

[B41] Iosefson O. Olivares A. O. Baker T. A. Sauer R. T. (2015). Dissection of axial-pore loop function during unfolding and translocation by a AAA+ proteolytic machine. Cell Rep. 12, 1032–1041. 10.1016/j.celrep.2015.07.007 26235618 PMC4536184

[B42] Jastrab J. B. Darwin K. H. (2015). Bacterial Proteasomes. Annu. Rev. Microbiol. 69, 109–127. 10.1146/annurev-micro-091014-104201 26488274 PMC4702487

[B43] Johnson E. S. Gonda D. K. Varshavsky A. (1990). Cis-trans recognition and subunit-specific degradation of short-lived proteins. Nature 346, 287–291. 10.1038/346287a0 2165217

[B44] Kim Y. C. Snoberger A. Schupp J. Smith D. M. (2015). ATP binding to neighbouring subunits and intersubunit allosteric coupling underlie proteasomal ATPase function. Nat. Commun. 6, 8520. 10.1038/ncomms9520 26465836 PMC4608255

[B45] Kirschner M. (1999). Intracellular proteolysis. Trends Cell Biol. 9, M42–M45. 10.1016/S0962-8924(99)01666-9 10611680

[B46] Kisselev A. F. Kaganovich D. Goldberg A. L. (2002). Binding of hydrophobic peptides to several non-catalytic sites promotes peptide hydrolysis by all active sites of 20 S proteasomes. Evidence for peptide-induced channel opening in the alpha-rings. J. Biol. Chem. 277, 22260–22270. 10.1074/jbc.M112360200 11927581

[B47] Kohler A. Cascio P. Leggett D. S. Woo K. M. Goldberg A. L. Finley D. (2001). The axial channel of the proteasome core particle is gated by the Rpt2 ATPase and controls both substrate entry and product release. Mol. Cell 7, 1143–1152. 10.1016/S1097-2765(01)00274-X 11430818

[B48] Kopito R. R. (2000). Aggresomes, inclusion bodies and protein aggregation. Trends Cell Biol. 10, 524–530. 10.1016/S0962-8924(00)01852-3 11121744

[B49] Kraut D. A. Israeli E. Schrader E. K. Patil A. Nakai K. Nanavati D.. (2012). Sequence- and species-dependence of proteasomal processivity. ACS Chem. Biol. 7, 1444–1453. 10.1021/cb3001155 22716912 PMC3423507

[B50] Kraut D. A. Prakash S. Matouschek A. (2007). To degrade or release: ubiquitin-chain remodeling. Trends Cell Biol. 17, 419–421. 10.1016/j.tcb.2007.06.008 17900906

[B51] Lam Y. A. Lawson T. G. Velayutham M. Zweier J. L. Pickart C. M. (2002). A proteasomal ATPase subunit recognizes the polyubiquitin degradation signal. Nature 416, 763–767. 10.1038/416763a 11961560

[B52] Lander G. C. Estrin E. Matyskiela M. E. Bashore C. Nogales E. Martin A. (2012). Complete subunit architecture of the proteasome regulatory particle. Nature 482, 186–191. 10.1038/nature10774 22237024 PMC3285539

[B53] Lander G. C. Martin A. Nogales E. (2013). The proteasome under the microscope: the regulatory particle in focus. Curr. Opin. Struct. Biol. 23, 243–251. 10.1016/j.sbi.2013.02.004 23498601 PMC3676703

[B54] Lee C. Schwartz M. P. Prakash S. Iwakura M. Matouschek A. (2001). ATP-dependent proteases degrade their substrates by processively unraveling them from the degradation signal. Mol. Cell 7, 627–637. 10.1016/S1097-2765(01)00209-X 11463387

[B55] Levitskaya J. Coram M. Levitsky V. Imreh S. Steigerwald-Mullen P. M. Klein G.. (1995). Inhibition of antigen processing by the internal repeat region of the Epstein-Barr virus nuclear antigen-1. Nature 375, 685–688. 10.1038/375685a0 7540727

[B56] Li D. Li H. Wang T. Pan H. Lin G. Li H. (2010). Structural basis for the assembly and gate closure mechanisms of the *Mycobacterium tuberculosis* 20S proteasome. EMBO J. 29, 2037–2047. 10.1038/emboj.2010.95 20461058 PMC2892373

[B57] Liu C. W. Corboy M. J. DeMartino G. N. Thomas P. J. (2003). Endoproteolytic activity of the proteasome. Science 299, 408–411. 10.1126/science.1079293 12481023 PMC3516294

[B58] Lowe J. Stock D. Jap B. Zwickl P. Baumeister W. Huber R. (1995). Crystal structure of the 20S proteasome from the archaeon *T. acidophilum* at 3.4 A resolution. Science 268, 533–539. 10.1126/science.7725097 7725097

[B59] Lupas A. N. Martin J. (2002). AAA proteins. Curr. Opin. Struct. Biol. 12, 746–753. 10.1016/S0959-440X(02)00388-3 12504679

[B60] Maillard R. A. Chistol G. Sen M. Righini M. Tan J. Kaiser C. M.. (2011). ClpX(P) generates mechanical force to unfold and translocate its protein substrates. Cell 145, 459–469. 10.1016/j.cell.2011.04.010 21529717 PMC3686100

[B61] Matyskiela M. E. Martin A. (2013). Design principles of a universal protein degradation machine. J. Mol. Biol. 425, 199–213. 10.1016/j.jmb.2012.11.001 23147216 PMC3546117

[B62] Medicherla B. Goldberg A. L. (2008). Heat shock and oxygen radicals stimulate ubiquitin-dependent degradation mainly of newly synthesized proteins. J. Cell Biol. 182, 663–673. 10.1083/jcb.200803022 18725537 PMC2518706

[B63] Moscovitz O. Tsvetkov P. Hazan N. Michaelevski I. Keisar H. Ben-Nissan G.. (2012). A mutually inhibitory feedback loop between the 20S proteasome and its regulator, NQO1. Mol. Cell 47, 76–86. 10.1016/j.molcel.2012.05.049 22793692

[B64] Nager A. R. Baker T. A. Sauer R. T. (2011). Stepwise unfolding of a beta barrel protein by the AAA+ ClpXP protease. J. Mol. Biol. 413, 4–16. 10.1016/j.jmb.2011.07.041 21821046 PMC3184388

[B65] Navon A. Goldberg A. L. (2001). Proteins are unfolded on the surface of the ATPase ring before transport into the proteasome. Mol. Cell 8, 1339–1349. 10.1016/S1097-2765(01)00407-5 11779508

[B66] Olivares A. O. Baker T. A. Sauer R. T. (2016). Mechanistic insights into bacterial AAA+ proteases and protein-remodelling machines. Nat. Rev. Microbiol. 14, 33–44. 10.1038/nrmicro.2015.4 26639779 PMC5458636

[B67] Orlowski M. Wilk S. (2000). Catalytic activities of the 20 S proteasome, a multicatalytic proteinase complex. Arch. Biochem. Biophys. 383, 1–16. 10.1006/abbi.2000.2036 11097171

[B68] Osmulski P. A. Hochstrasser M. Gaczynska M. (2009). A tetrahedral transition state at the active sites of the 20S proteasome is coupled to opening of the alpha-ring channel. Structure 17, 1137–1147. 10.1016/j.str.2009.06.011 19679091 PMC2746003

[B69] Park S. Li X. Kim H. M. Singh C. R. Tian G. Hoyt M. A.. (2013). Reconfiguration of the proteasome during chaperone-mediated assembly. Nature 497, 512–516. 10.1038/nature12123 23644457 PMC3687086

[B70] Peth A. Uchiki T. Goldberg A. L. (2010). ATP-dependent steps in the binding of ubiquitin conjugates to the 26S proteasome that commit to degradation. Mol. Cell 40, 671–681. 10.1016/j.molcel.2010.11.002 21095592 PMC3038635

[B71] Prakash S. Tian L. Ratliff K. S. Lehotzky R. E. Matouschek A. (2004). An unstructured initiation site is required for efficient proteasome-mediated degradation. Nat. Struct. Mol. Biol. 11, 830–837. 10.1038/nsmb814 15311270

[B72] Rabl J. Smith D. M. Yu Y. Chang S. C. Goldberg A. L. Cheng Y. (2008). Mechanism of gate opening in the 20S proteasome by the proteasomal ATPases. Mol. Cell 30, 360–368. 10.1016/j.molcel.2008.03.004 18471981 PMC4141531

[B73] Ravid T. Hochstrasser M. (2008). Diversity of degradation signals in the ubiquitin-proteasome system. Nat. Rev. Mol. Cell Biol. 9, 679–690. 10.1038/nrm2468 18698327 PMC2606094

[B74] Rechsteiner M. Hill C. P. (2005). Mobilizing the proteolytic machine: cell biological roles of proteasome activators and inhibitors. Trends Cell Biol. 15, 27–33. 10.1016/j.tcb.2004.11.003 15653075

[B75] Ripstein Z. A. Huang R. Augustyniak R. Kay L. E. Rubinstein J. L. (2017). Structure of a AAA+ unfoldase in the process of unfolding substrate. Elife 6:e25754. 10.7554/eLife.25754.001 28390173 PMC5423775

[B76] Rockel B. Jakana J. Chiu W. Baumeister W. (2002). Electron cryo-microscopy of VAT, the archaeal p97/CDC48 homologue from *Thermoplasma acidophilum*. J. Mol. Biol. 317, 673–681. 10.1006/jmbi.2002.5448 11955016

[B77] Rodriguez-Aliaga P. Ramirez L. Kim F. Bustamante C. Martin A. (2016). Substrate-translocating loops regulate mechanochemical coupling and power production in AAA+ protease ClpXP. Nat. Struct. Mol. Biol. 23, 974–981. 10.1038/nsmb.3298 27669037 PMC5467750

[B78] Rosenzweig R. Bronner V. Zhang D. Fushman D. Glickman M. H. (2012). Rpn1 and Rpn2 coordinate ubiquitin processing factors at proteasome. J. Biol. Chem. 287, 14659–14671. 10.1074/jbc.M111.316323 22318722 PMC3340268

[B79] Rubin D. M. Glickman M. H. Larsen C. N. Dhruvakumar S. Finley D. (1998). Active site mutants in the six regulatory particle ATPases reveal multiple roles for ATP in the proteasome. EMBO J. 17, 4909–4919. 10.1093/emboj/17.17.4909 9724628 PMC1170820

[B80] Ruschak A. M. Kay L. E. (2012). Proteasome allostery as a population shift between interchanging conformers. Proc. Natl. Acad. Sci. U.S.A. 109, E3454–E3462. 10.1073/pnas.1213640109 23150576 PMC3528551

[B81] Sauer R. T. Baker T. A. (2011). AAA+ proteases: ATP-fueled machines of protein destruction. Annu. Rev. Biochem. 80, 587–612. 10.1146/annurev-biochem-060408-172623 21469952

[B82] Schmidt M. Finley D. (2014). Regulation of proteasome activity in health and disease. Biochim. Biophys. Acta 1843, 13–25. 10.1016/j.bbamcr.2013.08.012 23994620 PMC3858528

[B83] Schmidt M. Lupas A. N. Finley D. (1999). Structure and mechanism of ATP-dependent proteases. Curr. Opin. Chem. Biol. 3, 584–591. 10.1016/S1367-5931(99)00013-7 10508673

[B84] Schweitzer A. Aufderheide A. Rudack T. Beck F. Pfeifer G. Plitzko J. M.. (2016). Structure of the human 26S proteasome at a resolution of 3.9 A. Proc. Natl. Acad. Sci. U.S.A. 113, 7816–7821. 10.1073/pnas.1608050113 27342858 PMC4948313

[B85] Sen M. Maillard R. A. Nyquist K. Rodriguez-Aliaga P. Presse S. Martin A.. (2013). The ClpXP protease unfolds substrates using a constant rate of pulling but different gears. Cell 155, 636–646. 10.1016/j.cell.2013.09.022 24243020 PMC3901371

[B86] Shabek N. Herman-Bachinsky Y. Buchsbaum S. Lewinson O. Haj-Yahya M. Hejjaoui M. (2012). The size of the proteasomal substrate determines whether its degradation will be mediated by mono- or polyubiquitylation. Mol. Cell 48, 87–97. 10.1016/j.molcel.2012.07.011 22902562

[B87] Shi Y. Chen X. Elsasser S. Stocks B. B. Tian G. Lee B. H.. (2016). Rpn1 provides adjacent receptor sites for substrate binding and deubiquitination by the proteasome. Science 351:aad9421. 10.1126/science.aad9421 26912900 PMC4980823

[B88] Smith D. M. Chang S. C. Park S. Finley D. Cheng Y. Goldberg A. L. (2007). Docking of the proteasomal ATPases' carboxyl termini in the 20S proteasome's alpha ring opens the gate for substrate entry. Mol. Cell 27, 731–744. 10.1016/j.molcel.2007.06.033 17803938 PMC2083707

[B89] Smith D. M. Fraga H. Reis C. Kafri G. Goldberg A. L. (2011). ATP binds to proteasomal ATPases in pairs with distinct functional effects, implying an ordered reaction cycle. Cell 144, 526–538. 10.1016/j.cell.2011.02.005 21335235 PMC3063399

[B90] Stinson B. M. Nager A. R. Glynn S. E. Schmitz K. R. Baker T. A. Sauer R. T. (2013). Nucleotide binding and conformational switching in the hexameric ring of a AAA+ machine. Cell 153, 628–639. 10.1016/j.cell.2013.03.029 23622246 PMC3674332

[B91] Striebel F. Hunkeler M. Summer H. Weber-Ban E. (2010). The mycobacterial Mpa-proteasome unfolds and degrades pupylated substrates by engaging Pup's N-terminus. EMBO J. 29, 1262–1271. 10.1038/emboj.2010.23 20203624 PMC2857465

[B92] Striebel F. Kress W. Weber-Ban E. (2009). Controlled destruction: AAA+ ATPases in protein degradation from bacteria to eukaryotes. Curr. Opin. Struct. Biol. 19, 209–217. 10.1016/j.sbi.2009.02.006 19362814

[B93] Takeuchi J. Chen H. Coffino P. (2007). Proteasome substrate degradation requires association plus extended peptide. EMBO J. 26, 123–131. 10.1038/sj.emboj.7601476 17170706 PMC1782366

[B94] Tanaka K. (2009). The proteasome: overview of structure and functions. Proc. Jpn. Acad. Ser. B Phys. Biol. Sci. 85, 12–36. 10.2183/pjab.85.12 19145068 PMC3524306

[B95] Thomsen N. D. Berger J. M. (2009). Running in reverse: the structural basis for translocation polarity in hexameric helicases. Cell 139, 523–534. 10.1016/j.cell.2009.08.043 19879839 PMC2772833

[B96] Tomko R. J. Jr. Hochstrasser M. (2011). Order of the proteasomal ATPases and eukaryotic proteasome assembly. Cell Biochem. Biophys. 60, 13–20. 10.1007/s12013-011-9178-4 21461838 PMC3256250

[B97] Tsvetkov P. Myers N. Moscovitz O. Sharon M. Prilusky J. Shaul Y. (2012). Thermo-resistant intrinsically disordered proteins are efficient 20S proteasome substrates. Mol. Biosyst. 8, 368–373. 10.1039/C1MB05283G 22027891

[B98] Tsvetkov P. Reuven N. Shaul Y. (2009). The nanny model for IDPs. Nat. Chem. Biol. 5, 778–781. 10.1038/nchembio.233 19841623

[B99] Turner G. C. Varshavsky A. (2000). Detecting and measuring cotranslational protein degradation *in vivo*. Science 289, 2117–2120. 10.1126/science.289.5487.2117 11000112

[B100] Unverdorben P. Beck F. Sledz P. Schweitzer A. Pfeifer G. Plitzko J. M.. (2014). Deep classification of a large cryo-EM dataset defines the conformational landscape of the 26S proteasome. Proc. Natl. Acad. Sci. U.S.A. 111, 5544–5549. 10.1073/pnas.1403409111 24706844 PMC3992697

[B101] Vabulas R. M. Hartl F. U. (2005). Protein synthesis upon acute nutrient restriction relies on proteasome function. Science 310, 1960–1963. 10.1126/science.1121925 16373576

[B102] van der Lee R. Lang B. Kruse K. Gsponer J. Sanchez de Groot N. Huynen M. A.. (2014). Intrinsically disordered segments affect protein half-life in the cell and during evolution. Cell Rep. 8, 1832–1844. 10.1016/j.celrep.2014.07.055 25220455 PMC4358326

[B103] Varshavsky A. (2011). The N-end rule pathway and regulation by proteolysis. Protein Sci. 20, 1298–1345. 10.1002/pro.666 21633985 PMC3189519

[B104] Verma R. McDonald H. Yates J. R. III. Deshaies R. J. (2001). Selective degradation of ubiquitinated Sic1 by purified 26S proteasome yields active S phase cyclin-Cdk. Mol. Cell 8, 439–448. 10.1016/S1097-2765(01)00308-2 11545745

[B105] Wang T. Darwin K. H. Li H. (2010). Binding-induced folding of prokaryotic ubiquitin-like protein on the Mycobacterium proteasomal ATPase targets substrates for degradation. Nat. Struct. Mol. Biol. 17, 1352–1357. 10.1038/nsmb.1918 20953180 PMC2988878

[B106] Wehmer M. Sakata E. (2016). Recent advances in the structural biology of the 26S proteasome. Int. J. Biochem. Cell Biol. 79, 437–442. 10.1016/j.biocel.2016.08.008 27498189

[B107] Wendler P. Ciniawsky S. Kock M. Kobe S. (2012). Structure and function of the AAA+ nucleotide binding pocket. Biochim. Biophys. Acta 1823, 2–14. 10.1016/j.bbamcr.2011.06.014 21839118

[B108] Yu Y. Smith D. M. Kim H. M. Rodriguez V. Goldberg A. L. Cheng Y. (2010). Interactions of PAN's C-termini with archaeal 20S proteasome and implications for the eukaryotic proteasome-ATPase interactions. EMBO J. 29, 692–702. 10.1038/emboj.2009.382 20019667 PMC2830694

[B109] Zhang M. Coffino P. (2004). Repeat sequence of Epstein-Barr virus-encoded nuclear antigen 1 protein interrupts proteasome substrate processing. J. Biol. Chem. 279, 8635–8641. 10.1074/jbc.M310449200 14688254

